# Impact of adhesive application errors on dentin bond strength of resin composite

**DOI:** 10.1080/26415275.2022.2138405

**Published:** 2022-11-07

**Authors:** Benjamin Michael Schärer, Anne Peutzfeldt

**Affiliations:** a Department of Restorative, Preventive and Pediatric Dentistry, School of Dental Medicine, University of Bern, Bern, Switzerland; b Department of Odontology, Faculty of Health and Medical Sciences, University of Copenhagen, Copenhagen, Denmark

**Keywords:** Adhesion, adhesive treatment, application errors, dentin bonding, resin composite

## Abstract

**Objectives**: To investigate the impact of adhesive application errors on dentin bond strength of resin composite.

**Material and Methods**: 165 extracted permanent human molars were ground to mid-coronal dentin. The dentin specimens were treated with one of three adhesive systems (OptiBond FL, Clearfil SE, Scotchbond Universal) either according to manufacturer’s instructions or with systematic errors in the application procedure and before application of resin composite (Filtek Z250). After storage (37 °C, 100% humidity, 24 h) shear bond strength (SBS) was measured and data analysed with either one-way ANOVA followed by Tukey tests (OptiBond FL, Scotchbond Universal, control groups) or Kruskal-Wallis followed by Wilcoxon tests (Clearfil SE). Finally, the failure mode of all specimens was assessed.

**Results**: With OptiBond FL and Clearfil SE omitted application (*p* ≤ 0.0001) as well as no evaporation (*p* ≤ 0.001) of the solvents in the primer significantly reduced the SBS. Omitted application of the adhesive, respectively the bond, had a negative influence on the SBS of Clearfil SE (*p* < 0.0001), but not of OptiBond FL (*p* = 0.776). With Scotchbond Universal, no evaporation of the solvents (*p* < 0.0001) as well as no light-cure (*p* = 0.0004) had a significant negative influence on the SBS. Using the adhesive systems according to manufacturer’s instructions, Clearfil SE achieved significantly lower SBS than OptiBond FL and Scotchbond Universal (*p* = 0.0027). Adhesive failure at the dentin surface was generally the most frequent failure mode observed.

**Conclusion**: All three adhesive systems tested were sensitive to application errors. For optimal result and longest possible durability of resin restorations, clinicians should strictly adhere to the manufacturer’s instructions.

## Introduction

To achieve solid bonding between hydrophobic resin composite and hydrophilic enamel and dentin, modern dentistry relies on adhesive systems. Whereas bonding to enamel has been effective and durable ever since the introduction of the enamel etching technique in the 1950s [1], reliable bonding to dentin turned out to be much more complicated to obtain. There are several reasons for this, such as the high organic content [2], the presence of outward fluid movements [3] and the tubular structure variations in dentin [4]. However, with their publication in 1991 on the role of the hybrid layer in creating adhesion *via* micromechanical retention, Nakabayashi et al. finally facilitated the development of more potent adhesive systems [5].

Current adhesive systems, which are supposed to eliminate the mentioned difficulties inherent to dentin, can be categorised into two main classes: the etch-and-rinse and the self-etch systems [6,7]. To summarize, in the etch-and-rinse systems first 32–37% phosphoric acid is applied which induces a superficial demineralisation of the dentin, whereby the collagen mesh is exposed [7]. This is followed by the application of a primer and subsequently by the application of an adhesive (three-step etch-and-rinse adhesive systems, e.g. OptiBond FL) or by the application a combined primer and adhesive (two-step etch-and-rinse adhesive system). The collagen mesh exposed by the phosphoric acid is thus infiltrated by monomers present in the primer/adhesive. Today, etch-and-rinse adhesive systems are still considered the gold standard as they are the oldest products on the market and can provide excellent results [8]. Due to the time-consuming and complex application of the etch-and-rinse adhesive systems, there has been a trend towards developing simplified adhesive systems, so-called self-etch adhesive systems [8]. These systems do not require application of phosphoric acid, as they comprise an acidic primer. For all systems however, the formation of the resin composite-dentin bond is the result of superficial dentin demineralisation with subsequent infiltration of resin monomers, which interlock micromechanically in the porosities after the polymerisation [9].

Each adhesive system is accompanied by the manufacturer’s instructions for use, which should be strictly adhered to during application. A survey in Denmark has shown that almost a quarter of the dentists questioned could not remember the correct application procedure of their adhesive system [10]. Thus, it is possible that in dental practice adhesive systems are not used according to the manufacturer’s instructions due to a lack of knowledge but also due to the time pressure in dentistry. A previous study investigated the effect on bond strength of incorrect use of six adhesive systems [11] and found the bond strength of resin composite to dentin to be significantly reduced by deviations from the manufacturer protocol. Twenty years have passed and in the meantime adhesive systems have undergone tremendous development. It seems relevant to investigate whether this continued development has resulted in less technique-sensitive adhesive systems.

Three adhesive systems with different complexity and time-consuming application were selected to represent current adhesive systems: a three-step etch-and-rinse adhesive system (OptiBond FL), a two-step self-etch adhesive system (Clearfil SE) and a one-component adhesive system (Scotchbond Universal). With seven application steps, the three-step etch-and-rinse adhesive system is the most complex and time-consuming to use. The two-step self-etch adhesive system requires six application steps, but because several steps are completed simultaneously (e.g. etching and application of the primer), considerably less time is required for the application. Finally, the one-component adhesive system has the simplest and quickest application procedure. The aim of this study was to investigate the impact of adhesive application errors in the three adhesive systems on dentin bond strength of resin composite. While the adhesive systems were applied according to the manufacturer’s instructions in the control groups, various application errors were committed in the experimental groups. Thus, the following null hypotheses were tested: (1) There are no differences in shear bond strength between the experimental groups and the corresponding control group. (2) There are no differences in shear bond strength between the control groups of the three adhesive systems.

## Material and methods

### Preparation of dentin specimens

A total of 165 dentin specimens were produced (*n* = 15 per group; 11 groups) from sound extracted permanent human molars obtained from a pooled biobank. The local ethical committee considers pooled biobanks as irreversibly anonymised and waives the necessity of previous ethical approval. The molars were cleaned with a scaler and curette and then ground parallel to the occlusal surface to the centre of the coronal dentin (Struers Labo-Pol 21; Struers, Ballerup, Denmark with Struers Silicon Carbide (SiC) abrasive paper, #220 and #500 with water cooling). It was ensured that the ground dentin surface had no residual enamel and no pulp opening. Subsequently, the roots were cut off using a water-cooled diamond saw (IsoMet Low Speed Saw; Buehler, Lake Bluff, IL, USA) and the molars were embedded in self-curing acrylic resin (Paladur; Heraeus Kulzer, Hanau, Germany) using stainless-steel moulds. After the acrylic resin had cured, the stainless-steel moulds were removed, and the dentin specimens were stored in the refrigerator (4 °C, 100% humidity) until the shear bond strength test specimens were produced.

### Preparation of shear bond strength specimens

The dentin specimens were retrieved from the refrigerator at least 1 h before use and stored in tap water at room temperature. The dentin of the specimens was re-roughened for 5 s with SiC abrasive paper #500 (Struers) to obtain a standardised smear layer. The SiC abrasive paper was replaced for each 10 specimens. Subsequently, the dentin specimens were randomly assigned to one of the 11 groups and again stored in tap water. Upon removal from the tap water, the dentin was carefully air-dried with a three-way syringe and a standardised adhesive area (*d* ≈ 2 mm) was defined using self-adhesive tape. The predefined adhesive area was then pre-treated with one of the adhesive systems OptiBond FL, Clearfil SE or Scotchbond Universal according to Table 1. The adhesive systems were used according to the manufacturers’ instructions. This implied that the primer and/or the adhesive of OptiBond FL and Scotchbond Universal were actively applied, while the primer and bond of the Clearfil SE Bond system were passively applied. For each adhesive system, Group 1 followed the instruction of the manufacturer, whereas Groups 2, 3 and 4 represented various errors in the application procedure. These errors consisted of either failing to apply the primer or the bond/adhesive, failure to evaporate the solvents in the primer or failure to light cure. Subsequently, a cylinder of resin composite (Filtek Z250) was bonded to the adhesive area using a split Teflon mold (inner diameter: 1.5 mm ≈ adhesive area: 1.8 mm^2^, height: 2 mm) mounted in a holding device. The resin composite cylinder was light-cured for 20 s (Demi, Kerr, Middleton, WI, USA; light power density: 1500 mW/cm^2^) and the specimen then stored in a lightproof box. After 5 min the Teflon mold was removed, and all specimens were returned to the lightproof boxes and kept at 37° C and 100% humidity for 24 h. All the materials used are listed in Table 2.

**Table 1.** Pre-treatment of dentin specimens (*n* = 15/group).

OptiBond FL											
		Time:			Time:			Time:			Time:
Group 1 (control)	Phosphoric acid 37%	15 s	Group 2	Phosphoric acid 37%	15 s	Group 3	Phosphoric acid 37%	15 s	Group 4	Phosphoric acid 37%	15 s
	Water-spray	15 s		Water-spray	15 s		Water-spray	15 s		Water-spray	15 s
	Air-dry	3 s		Air-dry	3 s		Air-dry	3 s		Air-dry	3 s
	OptiBond FL primer	15 s		OptiBond FL adhesive	–		OptiBond FL primer	15 s		OptiBond FL primer	15 s
	Air	3 s		Light-cure	20 s		OptiBond FL adhesive	–		Air	3 s
	OptiBond FL adhesive	–					Light-cure	20 s		Light-cure	20 s
	Light-cure	20 s									

Clearfil SE											
		Time:			Time:			Time:			Time:
Group 1 (control)	Clearfil SE Bond primer	–	Group 2	Clearfil SE Bond bond	–	Group 3	Clearfil SE Bond primer	–	Group 4	Clearfil SE Bond primer	–
	Waiting time	20 s		Air	3 s		Waiting time	20 s		Waiting time	20 s
	Air	3 s		Light-cure	20 s		Clearfil SE Bond bond	–		Air	3 s
	Clearfil SE Bond bond	–					Air	3 s		Light-cure	20 s
	Air	3 s					Light-cure	20 s			
	Light-cure	20 s									

Scotchbond Universal											
		Time:			Time:			Time:			
Group 1 (control)	Scotchbond Universal	20 s	Group 2	Scotchbond Universal	20 s	Group 3	Scotchbond Universal	20 s			
	Air	3 s		Light-cure	20 s		Air	3 s			
	Light-cure	20 s									

**Table 2.** Materials used.

Material	Composition according to the manufacturer	Manufacturer
Filtek Z250 (Lot: N783261)	Bis-GMA, UDMA, Bis-EMA, TEGDMA, zirconium/silica nonagglomerated particles	3M ESPE, St. Paul, MN, USA
OptiBond FL primer (Lot 7096704), adhesive (Lot 6921581)	*primer:* HEMA, GPDM, PAMM, ethanol, water, photoinitiator *adhesive:* TEGDMA, UDMA, GPDM, HEMA, Bis-GMA, filler, photoinitiator	Kerr, Orange, CA, USA
Clearfill SE primer (Lot 1E0348), bond (Lot 170616)	*primer:* MDP, HEMA, hydrophilic dimethacrylate, photoinitiator, water *bond:* MDP, Bis-GMA, HEMA, hydro- philic dimethacrylate, microfiller	Kuraray, Osaka, Japan
Scotchbond Universal (Lot 00729A)	MDP, Dimethacrylate resins, HEMA, Vitrebond™ Copolymer, filler, ethanol, water, initiators, silane	3M ESPE, St. Paul, MN, USA

Bis-EMA: Bisphenol-A polyethylenglycol dietherdimethacrylate; Bis-GMA: Bisphenol-A glycidylmethacrylate; GPDM: Glycerol phosphate dimethacrylate; HEMA: Hydroxyethylmethacrylate; MDP: 10-Methacryloyloxydecyl dihydrogen phosphate; METP: Methacryloyloxyethyl acid phosphate; PAMM: Phthalic acid monoethyl methacrylate; TEGDMA: Triethylene glycol dimethacrylate; UDMA: Urethane dimethacrylate.

### Bond strength testing and failure mode determination

After the 24 h storage, the specimens were subjected to bond strength testing performed in a universal testing machine (Zwick Z1.0 TN, Zwick, Ulm, Germany). The shear bond strength (SBS) specimens were loaded until fracture. The load was applied at a right angle to the resin composite cylinder at a crosshead speed of 1 mm/min. The strength required for fracture (F_max_ (N)) was determined and the SBS (MPa) was calculated as (F_max_ (N)/bonding area (mm^2^)). After bond strength testing, the failure mode of all specimens was assessed under a light microscope at a magnification of x30 (Leica ZOOM; Leica, Buffalo, NY, USA) and classified into the following categories: (1) cohesive failure in dentin, (2) adhesive failure at the dentin surface, (3) adhesive failure between adhesive system and resin composite, (4) cohesive failure in resin composite, (5) mixed failure.

### Statistical analysis

A Shapiro-Wilk’s test showed that the SBS values were normally distributed for the adhesive systems OptiBond FL and Scotchbond Universal, but not for Clearfil SE. Therefore, the effect of application mode was analysed separately for each adhesive system. The results obtained with OptiBond FL and Scotchbond Universal were analysed by parametric tests (one-way ANOVA and Tukey tests) and the results obtained with Clearfil SE with non-parametric tests (Kruskal-Wallis and Wilcoxon tests). The three control groups were compared with parametric tests (one-way ANOVA and Tukey test). The significance level was set at *p* = 0.05. All groups that were analysed with parametric tests are marked with capital letters in the boxplots and all groups that were analysed with non-parametric tests are marked with lower case letters. All statistical analysis was performed with R 3.3.3 (The R Project for Statistical Computing, Vienna, Austria; www.R-project.org). Failure modes after SBS testing were analysed descriptively.

## Results

**Figure 1.**
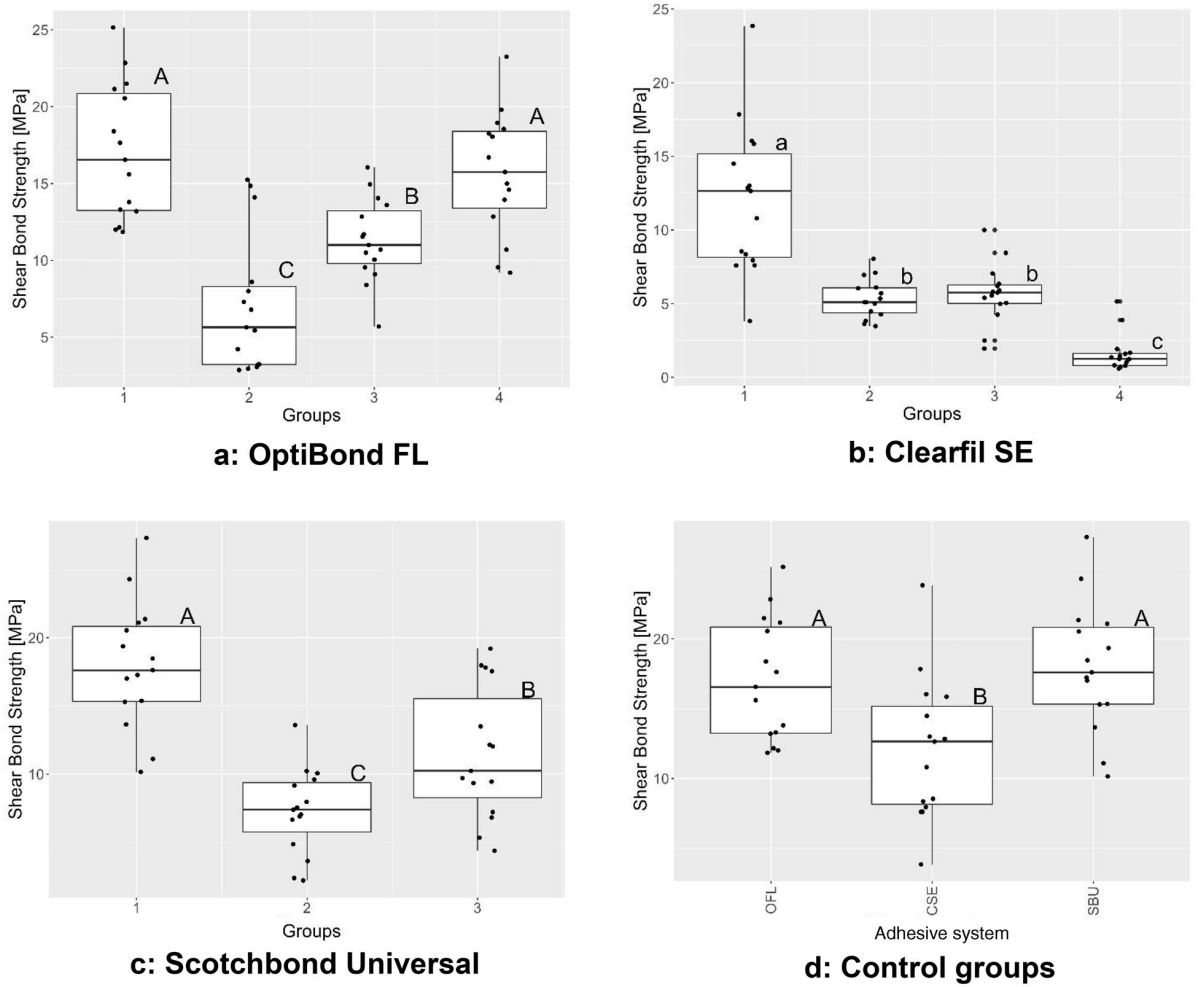
(a–d) Boxplots of the shear bond strength results (The horizontal line within each box represents the median value, the lower line represents the 1st quartile, and the top line represents the 3rd quartile. The whiskers extend vertically to within 1.5 of the IQR (= the distance between the first and the third quartiles). Same letters indicate no significant differences; different letters indicate significant differences between the SBS values.) CSE = Clearfil SE, OFL = OptiBond FL, SBU = Scotchbond Universal.

**Figure 2. (a-c):**
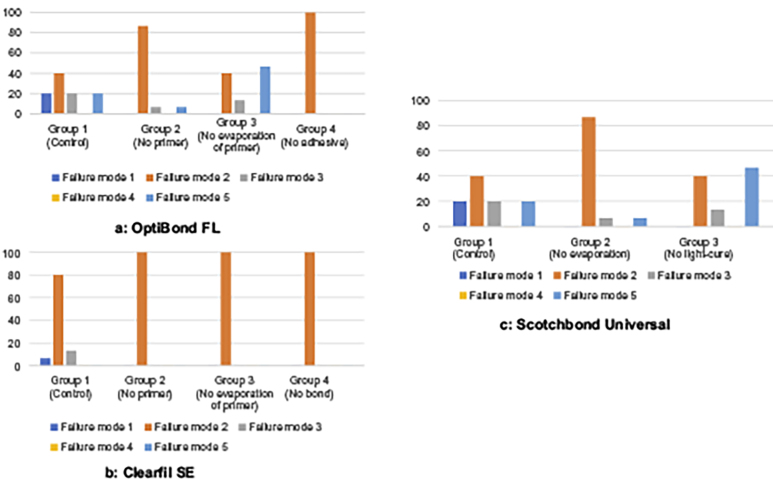
Failure mode 1: Cohesive in dentin; Failure mode 2: Adhesive at the dentin surface; Failure mode 3: Adhesive between adhesive system and resin composite; Failure mode 4: Cohesive in resin composite; Failure mode 5: Mixed (mixed type fracture pattern 1) to 4)).

The SBS results of all groups are presented in Table 3 as mean values and standard deviations while the results for each adhesive system and for the control groups are presented in Figure 1(a–d). The results of the failure modes are listed in Figure 2(a–c).

**Table 3.** Shear bond strength results (mean values and standard deviations; MPa). Mean

OptiBond FL		
Groups	Mean	Standard deviation
**1** (Control)	17.1^A^	4.4
**2** (No primer)	7.0^C^	4.4
**3** (No evaporation of primer)	11.3^B^	2.7
**4** (No adhesive)	15.7^A^	4.0

Clearfil SE		
Groups	Mean	Standard deviation
**1** (Control)	12.1^a^	5.1
**2** (No primer)	5.3^b^	1.3
**3** (No evaporation of primer)	5.7^b^	2.0
**4** (No bond)	1.6^c^	1.3

Scotchbond Universal		
Groups		Standard deviation
**1** (Control)	18.0^A^	4.6
**2** (No evaporation)	7.3^B^	3.1
**3** (No light-cure)	11.5^C^	4.8

Control groups		
Groups	Mean	Standard deviation
OptiBond FL	17.1^A^	4.4
Clearfil SE	12.1^B^	5.1
Scotchbond Universal	18.0^A^	4.6

For each of the four groups of comparison (OptiBond FL, Clearfil SE, Scotchbond Universal, Control groups), same letters indicate no significant difference, different letters indicate significant difference between groups. Capital letters = results analysed with parametric tests; lower case letters = results analysed with non-parametric tests.

### OptiBond FL

The one-way ANOVA found significant differences between the four groups i.e. between the four application procedures (*p* < 0.0001). The post-hoc tests found that failure to apply the primer (Group 2) significantly reduced the SBS compared to not only the control, but all other groups (Group 2 vs Group 1: *p* < 0.0001; Group 2 vs Group 3: *p* = 0.022; Group 2 vs Group 4: *p* < 0.0001). Similarly, but to a lesser degree, failure to evaporate the solvents in the primer (Group 3) significantly reduced the SBS compared to the control (Group 3 vs Group 1: *p* = 0.001) and to absence of adhesive (Group 3 vs Group 4: *p* = 0.019). In contrast, no difference was found between the control and the group in which the adhesive had not been applied (Group 1 vs Group 4; *p* = 0.776).

### Clearfil SE

The Kruskal-Wallis test found significant differences between the four groups i.e. between the four application procedures (*p* < 0.0001). The post-hoc tests found all deviations from the control (Group 1) to result in significantly lower SBS. Thus, failure to apply the primer (Group 2) or to evaporate the solvents in the primer (Group 3) had a negative, and similar, effect on the SBS compared to the control (Group 2 vs Group 1: *p* = 0.0001; Group 3 vs Group 1: *p* = 0.0002; Group 2 vs Group 3: *p* = 0.51). Similarly, and to an even higher degree, failure to apply the bond (Group 4) significantly reduced the SBS compared to the control (Group 4 vs Group 1: *p* < 0.0001) and compared to absence of primer (Group 4 vs Group 2: *p* < 0.0001) or failure to evaporate the solvents in the primer (Group 4 vs Group 3: *p* < 0.0001).

### Scotchbond Universal

The one-way ANOVA found significant differences between the three groups i.e. between the three application procedures (*p* < 0.0001). The post-hoc tests found both deviations from the control (Group 1) to result in significantly lower SBS. Thus, failure to evaporate the solvents in the adhesive (Group 2) not only reduced the SBS compared to the control (Group 1 vs Group 2: *p* < 0.0001) but also compared to failure to light-cure the adhesive (Group 3 vs Group 2: *p* = 0.02477). Similarly, but to a lesser degree, failure to light-cure the adhesive (Group 3) significantly reduced the SBS compared to the control (Group 3 vs Group 1: *p* = 0.0004).

### Control groups

The one-way ANOVA found a significant difference between the SBS values of the three control groups (*p* = 0.0027). The post-hoc tests found the SBS of Clearfil SE to be significantly lower than that of OptiBond FL (*p* = 0.0165) and Scotchbond Universal (*p* = 0.0037), while there was no significant difference between Scotchbond Universal and OptiBond FL (*p* = 0.8487).

### Failure modes

With the adhesive system OptiBond FL, adhesive failure at the dentin surface (failure mode 2) was the most frequently observed failure mode in all groups, except in Group 3. In this group mixed failure (failure mode 5) was the most frequent. With Clearfil SE, adhesive failure at the dentin surface (failure mode 2) was either the only or the most frequent failure mode. Finally, with Scotchbond Universal, mixed failure (failure mode 5) was the most frequent failure mode in Group 1. In contrast, the other two groups showed mostly adhesive failures at the dentin surface (failure mode 2).

## Discussion

This *in vitro* study investigated the impact of errors in the application procedure of three adhesive systems (OptiBond FL, Clearfil SE and Scotchbond Universal) on dentin bond strength of resin composite. Significant decreases in the dentin bond strength were observed for all three adhesive systems as a consequence of application errors, meaning that the first null hypothesis (there are no differences in shear bond strength between the experimental groups and the corresponding control group) cannot be accepted.

For the OptiBond FL adhesive system, failure to apply the primer (Group 2) led to a significant drop in bond strength and to a higher occurrence of adhesive failures at the dentin surface compared to the control. Due to the absence of the primer, the dentin with its collagen fibre network was not prepared for the hydrophobic monomers present in the subsequently applied adhesive. Consequently, it can be assumed that no hybrid layer was formed which explains the poor bond strength obtained [7,12]. Based on an understanding of the bonding mechanisms, it is surprising that a previous study found no negative effect on the dentin bond strength in the absence of the primer [13]. Failure to allow the water and ethanol solvents in the OptiBond FL primer to evaporate (Group 3) also led to significantly lower bond strength albeit not to the same drastic degree as complete absence of primer. Any solvents retained in the collagen network due to incomplete evaporation may have hindered complete infiltration of the monomers, and thus formation of a proper hybrid layer [14]. Retention of the solvents in the hybrid layer may also have hampered polymerization of the adhesive monomers leading to lower bond strength. The high prevalence of adhesive failures at the dentin surface and mixed failures may be interpreted as signs of residual moisture on the bonding surface and inhibited light-curing. Surprisingly, failure to apply the OptiBond FL adhesive (Group 4) had no negative effect on the dentin bond strength compared to the control group. A possible explanation is the difference between the clinical and the *in vitro* setting. In the two groups, i.e. with or without application of the adhesive, the phosphoric acid etching exposed the dentinal tubules and the collagen fibre network, allowing the primer to penetrate these structures and subsequently interact with the collagen fibre network [15]. The monomers of the adhesive used in the following step then copolymerized with the monomers of the primer. Upon curing and in the clinical setting, the adhesive thus prevents a negative influence of intrinsic moisture due to the increased dentin permeability of vital teeth following phosphoric acid etching, whereby moisture leaks into the cavity *via* the dentin tubules due to the pulp pressure [16]. However, in this study, extracted, devitalised teeth were used, so no intrinsic humidity was generated by the pulp pressure to negatively influence bonding. As regards failure mode the control group showed a heterogeneous distribution of failure modes, whereas omission of the adhesive resulted exclusively in adhesive failures at the dentin surface, showing that the dentin-adhesive interface was the ‘weakest link’. Based on this indication of a difference in bonding mechanism and on *in vivo* conditions being more challenging that the *in vitro* setting applied in the present study, we urgently recommend using the primer as well as the adhesive and to meticulously follow the manufacturer’s instructions.

For the Clearfil SE adhesive system, all three deviations from the instructions for use led to significantly lower bond strengths and to 100% adhesive failures at the dentin surface. Failure to apply the bond component (Group 4) caused the highest drop in bond strength. The fact that omission of the bond had such a marked effect on bond strength could reflect that the etching pattern after pretreatment with a self-etching primer, such as the Clearfil SE primer, differs from the etching pattern created by the phosphoric acid pretreatment of etch-and-rinse adhesive systems. The self-etch adhesive systems have been found to produce less defined etching patterns [17], and it could be speculated that the subsequently applied bond component would then play a more decisive role in creating a stable bond between dentin and resin composite. Failure to apply the Clearfil SE primer (Group 2) also had a negative effect on the bond strength, but to a lesser degree than omission of the bond (Group 4). With self-etch adhesive systems, etching of the tooth structure is obtained by the acidic primer. Omission of primer application may have weakened the dentin bonding in two ways. First, etching of the dentin by the acid phosphate monomer 10-methacryloyloxydecyl dihydrogen phosphate (MDP), which has been shown to be essential for providing a stable bond between resin composite and tooth structure [18], did not take place. Secondly, the absence of MDP also meant that the hydrophobic interactions between the hydrophobic MDP and the hydrophobic collagen surface did not occur [19]. The absence of etching and of interaction prevented optimal micromechanical retention of the resin composite to the dentin, which is the most important mechanism for a stable bonding between these two components [20]. Failure to evaporate the solvents in the Clearfil SE primer (Group 3) lowered the bond strength to the same degree as failure to apply the primer. As discussed for Optbond FL, residual moisture from the non-evaporated water solvent in the Clearfil SE primer could have inhibited the curing of the bond component as well as the curing of the resin composite. However, it is noteworthy that for Clearfil SE, failure to apply the primer and failure to evaporate the solvents in the primer had statistically similar detrimental effect on the bond strength, whereas for OptiBond FL, failure to apply the primer had a more detrimental effect than ‘simply’ failing to evaporate it. The fact that the two adhesive systems reacted differently could be explained by the circumstance that Clearfil SE primer uses only water as solvent, whereas the OptiBond FL primer uses both water and ethanol. Because ethanol is more volatile than water, absence of the evaporating step might be less crucial.

For the Scotchbond Universal adhesive system, both deviations from the instructions for use led to a decrease in bond strength and an increase in the percentage of adhesive failures at the dentin surface. Failure to evaporate the solvents in the adhesive (Group 2) had the most detrimental effect. Like OptiBond FL, Scotchbond Universal contains ethanol as well as water as solvents. Lack of the evaporation step may be assumed to result in increased residual moisture on the dentin surface. This moisture has been found to not affect the thickness of the adhesive nor of the hybrid layer, but to shorten the resin tags [21]. A number of studies have shown that shortened resin tags can negatively affect the performance of Scotchbond Universal [21,22]. In contrast, other studies have found no negative impact of increased residual moisture [23,24]. Failure to light-cure (group 3) also led to lower bond strength. The light-curing of an adhesive or a resin composite converts the monomers to stable high-molecular weight polymers [25], and the higher the degree of conversion, the better the mechanical properties [26]. Although some degree of polymerization of the adhesive might have occurred during the subsequent light-curing of the resin composite, the layer thickness of the composite of 2 mm, is likely to have reduced the degree of polymerization significantly [27,28] and thus to have caused a reduction in bond strength.

Finally, a comparison between the three adhesive systems when they had all been applied according to the manufacturers’ instructions found significant differences and thus, the second null hypothesis (that there are no differences in shear bond strength between the control groups of the adhesive systems) cannot be accepted. OptiBond FL and Scotchbond Universal resulted in significantly higher bond strength than did Clearfil SE. Whereas these results are in contrast with a number of previous studies in which Clearfil SE gave similar bond strength as OptiBond FL [29] and Scotchbond Universal [11], other studies corroborate the present results, i.e. that Clearfil SE gave significantly lower bond strength than Scotchbond Universal [30,31]. The fact that the findings and conclusions vary between the studies might be explained on the one hand by different operators and their experience in dentistry and on the other hand by different study setups (e.g. type of bond strength test used and the storage conditions of the specimens).

Based on this *in vitro* study of the bond strength of resin composite to dentin, it can be concluded that all three adhesive systems evaluated, i.e. a three-step etch-an-rinse, a two-step self-etch, and a one-component adhesive system, and of varying complexity and time on the marked, were sensitive to application errors. While the present study focused on grave errors such as failing to apply a primer or an adhesive, numerous other errors can occur in the clinical application, which could potentially hamper the adhesion of the restoration e.g. contamination of the bonding surfaces, over-drying the dentin after phosphoric acid etching and non-compliance with application or light-curing times. To conclude, for an optimal result and the longest possible durability of resin restorations, clinicians should strictly adhere to the instructions for use advocated by the respective manufacturers.
